# BeatWalk: Personalized Music-Based Gait Rehabilitation in Parkinson’s Disease

**DOI:** 10.3389/fpsyg.2021.655121

**Published:** 2021-04-26

**Authors:** Valérie Cochen De Cock, Dobromir Dotov, Loic Damm, Sandy Lacombe, Petra Ihalainen, Marie Christine Picot, Florence Galtier, Cindy Lebrun, Aurélie Giordano, Valérie Driss, Christian Geny, Ainara Garzo, Erik Hernandez, Edith Van Dyck, Marc Leman, Rudi Villing, Benoit G. Bardy, Simone Dalla Bella

**Affiliations:** ^1^Department of Neurology, Beau Soleil Clinic, Montpellier, France; ^2^EuroMov Digital Health in Motion, University of Montpellier, IMT Mines Alès, Montpellier, France; ^3^LIVELab, McMaster University, Hamilton, ON, Canada; ^4^Department of Epidemiology and Biostatistics, Beau Soleil Clinic, Montpellier, France; ^5^INSERM, Clinical Investigation Centre (CIC) 1411, University Hospital of Montpellier, Montpellier, France; ^6^Clinical Research and Epidemiology Unit, Medical Information Department, CHU Montpellier, University of Montpellier, Montpellier, France; ^7^Department of Neurology, University Hospital of Montpellier, Montpellier, France; ^8^Neuroengineering Area, Health Division, TECNALIA, Basque Research and Technology Alliance (BRTA), Donostia-San Sebastian, Spain; ^9^Department of Musicology, Institute for Psychoacoustics and Electronic Music (IPEM), Ghent University, Ghent, Belgium; ^10^Department of Electronic Engineering, Maynooth University, Maynooth, Ireland; ^11^International Laboratory for Brain, Music, and Sound Research (BRAMS), Montreal, QC, Canada; ^12^Department of Psychology, University of Montreal, Montreal, QC, Canada; ^13^Centre for Research on Brain, Language and Music, Montreal, QC, Canada; ^14^Department of Cognitive Psychology, University of Economics and Human Sciences in Warsaw, Warsaw, Poland

**Keywords:** Parkinson’s disease, cueing, rhythmic auditory stimulation, physical activity, gait rehabilitation, precision medicine BeatWalk

## Abstract

Taking regular walks when living with Parkinson’s disease (PD) has beneficial effects on movement and quality of life. Yet, patients usually show reduced physical activity compared to healthy older adults. Using auditory stimulation such as music can facilitate walking but patients vary significantly in their response. An individualized approach adapting musical tempo to patients’ gait cadence, and capitalizing on these individual differences, is likely to provide a rewarding experience, increasing motivation for walk-in PD. We aim to evaluate the observance, safety, tolerance, usability, and enjoyment of a new smartphone application. It was coupled with wearable sensors (BeatWalk) and delivered individualized musical stimulation for gait auto-rehabilitation at home. Forty-five patients with PD underwent a 1-month, outdoor, uncontrolled gait rehabilitation program, using the BeatWalk application (30 min/day, 5 days/week). The music tempo was being aligned in real-time to patients’ gait cadence in a way that could foster an increase up to +10% of their spontaneous cadence. Open-label evaluation was based on BeatWalk use measures, questionnaires, and a six-minute walk test. Patients used the application 78.8% (±28.2) of the prescribed duration and enjoyed it throughout the program. The application was considered “easy to use” by 75% of the patients. Pain, fatigue, and falls did not increase. Fear of falling decreased and quality of life improved. After the program, patients improved their gait parameters in the six-minute walk test without musical stimulation. BeatWalk is an easy to use, safe, and enjoyable musical application for individualized gait rehabilitation in PD. It increases “walk for exercise” duration thanks to high observance.

**Clinical Trial Registration**: ClinicalTrials.gov Identifier: NCT02647242.

## Introduction

In patients with Parkinson’s disease (PD), physical activity has positive effects on strength, balance, gait and quality of life (Lauze et al., 2016; Porta et al., 2018). Instead of engaging in physical activity, however, patients report being one-third less active compared to healthy older adults (van Nimwegen et al., 2011). Reduced physical activity can even initiate a cycle of deconditioning and progressive disability, independent of latent disease progress, worsening motor and non-motor symptoms of PD (van Nimwegen et al., 2011).

Different factors such as perceived self-efficacy and enjoyment influence patients’ involvement with physical activity (Urell et al., 2019). In particular, enjoyment plays a critical role in maintaining physical activity over time and in improving the quality of life (Urell et al., 2019). The clinical setting in which patients perform physical activity may not always be very motivating. Hence, there is an urgent need for alternative and more motivating strategies to promote physical activity in PD. Here we propose that walking with music, while exercising outdoor in a non-clinical setting, is a viable, enjoyable, and motivating alternative. Our approach is coupled with the use of mobile technologies capable of providing performance feedback, an important aspect of motivation in physical activity (Ginis et al., 2016) seldom proposed to patients.

Music and other rhythmic auditory cues have immediate as well as longer-term beneficial effects on gait parameters in PD (Thaut et al., 1996; Ghai et al., 2018). Yet, PD symptoms are highly heterogeneous, and the effects of music stimulation can vary significantly among studies (de Dreu et al., 2012) and patients (Dalla Bella et al., 2017; Cochen De Cock et al., 2018). We recently showed that the variable response to rhythmic cues is linked to inter-individual differences in rhythmic abilities (Cochen De Cock et al., 2018; Dalla Bella et al., 2018). Indeed, difficulties in rhythm perception and synchronization common in PD (Grahn and Brett, 2009; Benoit et al., 2014; Bienkiewicz and Craig, 2015) are likely to negatively modulate otherwise positive effects of musical rhythm on movement (Dalla Bella et al., 2017; Cochen De Cock et al., 2018; Dalla Bella, 2020).

There is growing evidence that individualized and interactive music or rhythm stimulation strategies that foster spontaneous entrainment hold some promise in improving gait performance (Hove et al., 2012; Dotov et al., 2019). In these strategies, the stimulus is modified in real-time so that its beat is dynamically aligned to the patient’s steps. Spontaneous entrainment fosters a more natural, paced walking experience. More importantly, it avoids the need for instructed purposeful beat-step synchronization, a solution that is not always beneficial (Ready et al., 2019).

In this study, we tested a new personalized music-based gait rehabilitation protocol embedded in a smartphone application, called BeatWalk. The stimulation is interactive and aims to find the optimal compromise between individual patient’s capacities and stimulus features, by building on coupled-oscillators modeling of gait temporal dynamics (Damm et al., 2019; Dotov et al., 2019).

BeatWalk allows the patients to walk outdoor, with performance feedback, while listening to step-synchronized music of various genres. We hypothesized that the ratio benefits/risks of BeatWalk use is positive. To test this we measured observance, safety, tolerance, usability, and enjoyment as in a phase 2 clinical trial. We also examined how BeatWalk can promote physical activity.

## Participants and Methods

### Participants

Forty-five patients with PD (aged 65 ± 9; 25 males) exhibiting gait disorders but able to walk unaided (Item 10 of the MDS-UPDRS-III ≥1 and <3; Martinez-Martin et al., 2013), without freezing, were recruited from the Department of Neurology of the Beau Soleil Clinic and the Regional University Hospital of Montpellier (France).

Diagnosis was established according to the Queen Square Brain Bank criteria (Hughes et al., 1992). Six participants interrupted the study without using BeatWalk, two because of inability to use the application from the beginning, and four because of inability to walk for 30 min. They did not differ from the 39 other patients on age, sex, disease severity, treatment, and gait measures. Participants were kept on their usual medications during the evaluation. Disease severity was moderate (MDS-UPDRS-III = 26.71 ± 12.06, Hoehn and Yahr stage = 2.4 ± 0.5). Disease duration was 7.6 ± 4.6 years and age at onset 57.4 ± 10.2 years. Levodopa equivalent daily dose was 701 ± 441 mg (Tomlinson et al., 2010) and 66.6% of the patients received dopamine agonists, 37.7% monoamine oxydase inhibitors, 9% entacapone and none had anticholinergics.

The study was approved by the National Ethics Committee (CPP Sud Méditérannée III, Nîmes, France, ID-RCB: 2015-A00531-48). All participants gave written informed consent before participating.

## Procedure

### Rehabilitation Program

Patients were asked to walk alone outside in a safe environment (with no cars, without crossing roads, and on regular ground) while listening to musical stimuli for 30 min, five times a week, for 4 weeks. During each session, they could stop up to four times and for maximum of combined 10 min.

### BeatWalk Application (Figure 1)

**Figure 1:**
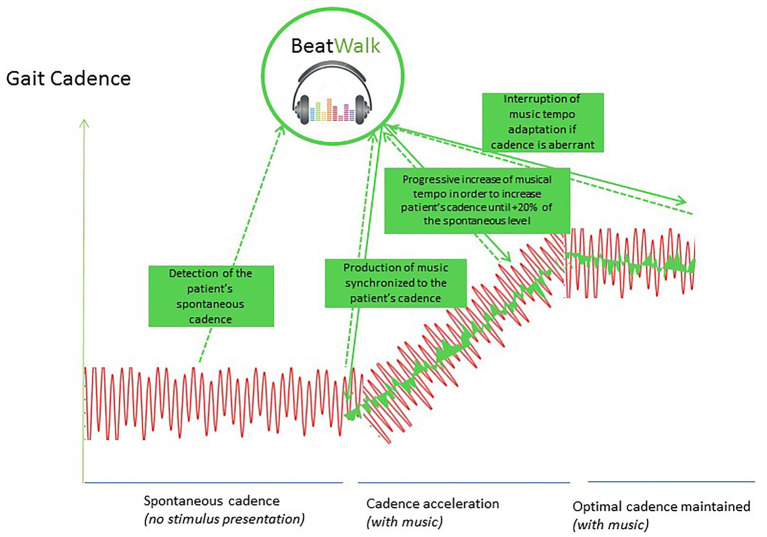
BeatWalk operation: (1) first BeatWalk is silent and detects the patient’s spontaneous gait cadence and phase, (2) BeatWalk generates music with a tempo synchronized to the patient’s spontaneous cadence, (3) BeatWalk modifies subliminally the tempo of the music (changing the peace of music if necessary), to increase it progressively at maximum +20% of the patients spontaneous cadence, (4) BeatWalk maintains the tempo of the music in the targeted cadence range.

BeatWalk includes a smartphone application (Garzo et al., 2018) that modifies the tempo of the music in order to induce spontaneous mutual synchronization with patients’ gait (Dotov et al., 2019) and ankle-worn sensors. The procedure implemented in BeatWalk is illustrated in Figure 1. The music tempo was updated in real-time to match the patient’s gait cadence calculated from the five most recent footfalls. Footfalls were detected by streaming angular velocities and accelerations of the lower limbs measured by inertial sensors to the smartphone. We selected the adaptation parameters such that the stimulus was interactive, but also left enough space for the participants to adapt to it, thus encouraging mutual synchronization. Using the participant’s 1 min pre-test at each session as a baseline, we set the intrinsic tempo of the stimulus so that the effective compromise tempo where stimulus and gait met would be anywhere between 100 and 120% of baseline depending on the given participant’s capacity to increase his or her cadence (Dotov et al., 2019).

The custom software played auditory tracks prepared in advance and paired with meta-information files annotated with beat times. We sorted 285 pieces of music in six genres (disco, pop, soft pop, pop rock, instrumental, and variety). Patients had to choose at least two genres for each session. The program applied online stimulus adaptation. To this end, a phase vocoder time-stretching algorithm modulated the song tempo without producing audible deformations of pitch (Moens et al., 2014). Time-stretching was controlled by the interactive stimulus oscillator using step phase and cadence computed online from the latest detected footfalls.

### BeatWalk Interface (Figure 2)

**Figure 2:**
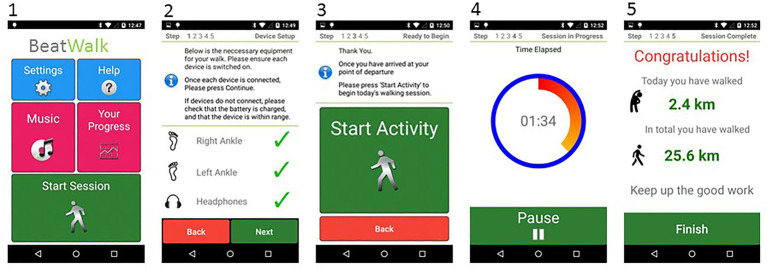
BeatWalk interface: (1) starting screen, (2) sensors connection check, (3) session starting, (4) session progression, (5) encouraging feedback with performances.

The application was designed (1) to increase the patient’s motivation to walk, and, (2) to provide clear and simple instructions ensuring usability for patients with PD. The patient could choose the musical genre for each session. Encouraging feedback about session progress was provided during the session. At the end of each session, feedback was given about gait performance (e.g., gait speed, distance traveled) in the latest and all previous sessions (Garzo et al., 2018).

## Evaluations

Neurological and neuropsychological evaluations and gait measurements were performed before and after the 4-week rehabilitation program.

### Neurological and Neuropsychological Evaluation

Demographic characteristics and medical history were collected in a preliminary interview. Severity of the disease was evaluated on the Hoehn and Yahr scale (Hoehn and Yahr, 1967) and the revised Movement Disorder Society-Unified Parkinson’s Disease Rating Scale (MDS-UPDRS; Martinez-Martin et al., 2013). The levodopa equivalent daily dose was calculated (Tomlinson et al., 2010). Self-evaluation of the risk of falls was provided by the patients using the Falls Self-Efficacy Scale Score (Tinetti et al., 1990). Balance was evaluated using the Mini-BESTest (Franchignoni et al., 2010).

We evaluated global cognitive functioning with the Montreal Cognitive Assessment (Dalrymple-Alford et al., 2010), depressive symptoms, with the Beck Depression Inventory (Beck et al., 1961), anxiety with the Parkinson Anxiety Scale (Leentjens et al., 2014), apathy with the Lille Apathy Rating Scale (Dujardin et al., 2013), fatigue with Fatigue severity scale (Krupp et al., 1989), and quality of life using with EQ5-D (Schrag et al., 2000).

### Safety and Tolerance

All patients completed a daily survey about their number of falls and about their fatigue and pain with visual sliding scales during the 2 weeks before rehabilitation (baseline evaluation) and during the 4 weeks of the program. They were also asked about other side effects.

### Training Program Observance, Usability, and Enjoyment

Observance was calculated as the effective amount of time the program was used, expressed as the percentage of the prescribed use duration (10 h on total). Usability was evaluated using a scale proposed to evaluate smartphone interventions (Ben-Zeev et al., 2014). We also measured physical activity enjoyment associated with each session using the Physical Activity Enjoyment Scale (Mullen et al., 2011). Patients were asked to rate “How do you feel at the moment about the physical activity you have been doing” using a seven-point bipolar rating scale. Higher PACES scores reflect greater levels of enjoyment.

### Physical Activity Evaluation

We explored physical activity before and during the program using a questionnaire developed for the Community Healthy Activities Model Program for Seniors (CHAMPS; Stewart et al., 2001). This questionnaire assesses the weekly frequency and duration of various physical activities typically undertaken by older adults and allows an estimation of physical activity caloric expenditure/week. This caloric expenditure was calculated for all specified physical activities, including those of light intensity and for activities of at least moderate intensity (MET value ≥ 3.0).

### Gait Measurements

Gait spatiotemporal parameters were recorded during a six-minute walk test *via* sensors (inertial measurement units including 3D accelerometers and gyroscopes, MobilityLab, APDM Inc., Portland) strapped over the feet and anterior side of the left and right tibia, and sternum. Each patient was assessed at the same time of day in “on” condition, ~1 h after drug intake, to control for variations due to the drug cycle.

### Statistical Analyses

Categorical variables were presented as percentages and quantitative variables as means and standard deviations (SDs). Results before and after the rehabilitation program were compared using a Wilcoxon signed-rank test. Chi-squared tests or Fisher’s exact tests were used for categorical ones. Significance level was set at *p* < 0.05.

## Results

### Observance

Patients used the application 78.8% (±28.2) of the prescribed duration. Only 7.7% of the patients (*n* = 3) used BeatWalk <25% of the prescribed duration and 48.7% (*n* = 19) used it more than 90%. Patients performed on average 15.9 ± 5.8 sessions (range: 1–20), out of the 20 sessions prescribed.

The average session duration was 29.71 ± 1.10 min (instructed duration: 30 min) and the mean distance traveled was 2.42 ± 0.53 km per session. The overall distance traveled by the patients was 39.2 ± 17.3 km. Patients with reduced observance of the program (lower than 50%, *n* = 8), as compared to other patients in the group (*n* = 31), showed greater fear of falling (FSESS: 34.00 ± 11.94 vs. 25.71 ± 7.67, *p* = 0.05), impairment in motor daily living (MDS-UPDRS-II: 14.86 ± 5.27 vs. 8.71 ± 6.46, *p* = 0.02), and lower quality of life (EQ5D: 9.14 ± 1.68 vs. 7.86 ± 1.27, *p* = 0.05) before the program. They did not differ at the 6-min walk test at baseline for speed, stride length, or cadence.

### Safety and Tolerance

The number of falls per week did not increase due to BeatWalk use and longer times spent walking. On the contrary, the patients tended to show a lower number of falls during the rehabilitation program than they typically experienced in a week (0.22 ± 0.76 vs. 0.11 ± 0.46, *p* = 0.07). We also observed a reduction in the number of fallers during the last 2 weeks of the rehabilitation program compared to the 2 weeks of baseline (8.9 vs. 11.1%, *p* = 0.01).

During BeatWalk use the patients reported a reduction of pain relative to the baseline (2.80 ± 1.71 vs. 2.21 ± 1.57, *p* = 0.02).

Fatigue while using BeatWalk did not change compared to baseline, on visual sliding scale (3.22 ± 2.13 vs. 3.99 ± 1.95, *p* = 0.3) and on fatigue severity scale (32.65 ± 15.87 vs. 30.52 ± 13.53, *p* = 0.5).

Patients did not report other side effects.

### Usability and Enjoyment

**Figure 3:**
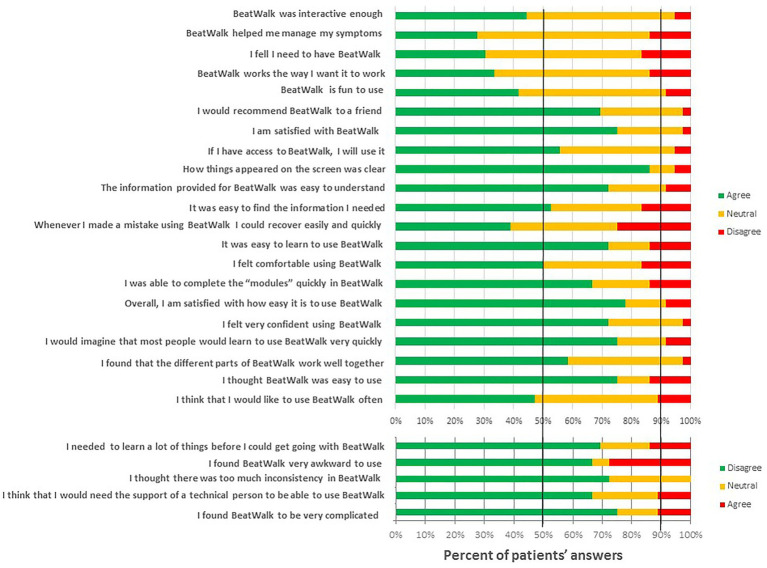
Evaluation by the patients of different parameters of usability. In green responses favorable to BeatWalk, in orange, neutral, and in red unfavorable.

Patients’ responses to the different items in the acceptability/usability tests are reported in Figure 3. The number of patients with positive responses on usability items was much higher than with negative responses (60.5 vs. 10.8%, *p* < 0.001).

The score obtained in the Physical Activity Enjoyment Scale was 29.21 ± 8.61 points (min 8 and max 56) after 1 week of use of BeatWalk and 90.3% of the patients experienced “sufficient levels of joy” (mean over 24; Mullen et al., 2011). Interestingly, this score and percentage did not change over time and remained as high after 1 month of use (respectively, 28.33 ± 8.46, *p* = 0.2, and 92.0%).

Quality of life and Self-evaluation of fall risk are reported in Table 1. Fear of falling decreased and the quality of life increased after the rehabilitation program using BeatWalk. However, it did not modify the severity of the disease or the neuropsychological and balance evaluations.

**Table 1:** Clinical characteristics of patients with Parkinson’s disease (PD) before and after treatment.

	Before rehabilitation program	After rehabilitation program	*P*
Number of participants	39	39	
**Parkinson’s disease evaluation**			
Hoehn and Yahr	2.4 ± 0.5	2.5 ± 0.5	0.40
MDS-UPDRS-I	9.04 ± 6.27	7.67 ± 5.99	0.46
MDS-UPDRS-II	9.44 ± 6.50	9.71 ± 8.35	0.54
MDS-UPDRS-III	26.71 ± 12.06	22.87 ± 15.39	0.30
MDS-UPDRS-IV	4.11 ± 3.66	4.24 ± 3.66	0.67
**Balance**			
Falls self-efficacy score	26.87 ± 9.03	24.73 ± 7.86	0.05
Mini Best test	24.13 ± 3.12	24.73 ± 2.62	0.18
**Quality of life**			
EQ5D	7.89 ± 1.42	7.59 ± 1.57	0.03
EQ5D EVA	65.78 ± 16.99	66.76 ± 15.56	0.57
**Psychological evaluation**			
Depression (BDI)	11.53 ± 6.74	11.81 ± 8.43	0.91
Anxiety (PAS)	12.82 ± 7.16	12.84 ± 8.10	0.57
Apathy (LARS)	−7.3 ± 4.6	−9.0 ± 3.4	0.02
**6-min test**			
Distance	452.66 ± 75.39	470.29 ± 60.11	0.01
Cadence (steps/min)	118.01 ± 11.88	121.08 ± 10.13	0.01
Velocity (m/s)	1.28 ± 0.20	1.32 ± 0.17	<0.01
Stride length (m)	1.29 ± 0.13	1.31 ± 0.15	0.04
Asymmetry index (PCI, %)	5.42 ± 2.28	5.41 ± 2.46	0.49

MDS-UPDRS: Movement Disorder Society-Unified Parkinson’s Disease Rating Scale, EQ5D: European Quality of life-5 Domains, BDI: Beck Depression Inventory, PSA: Parkinson Anxiety Scale, LARS: Lille Apathy Rating Scale, PCI: Phase Coordination Index.

### Physical Activity Evaluation

The frequency of physical activities and the associated caloric expenditures before and after the rehabilitation program are reported in Table 2. An increase was observed in the frequency of activities with at least moderate intensity and “walk for exercise.” Caloric expenditure linked to “walk for exercise” and fast walk activities increased during the rehabilitation program.

**Table 2:** Physical activity of patients with PD before and during BeatWalk rehabilitation program: caloric expenditure and frequency of exercises measured with the Community Healthy Activities Model Program for Seniors (CHAMPS) questionnaire.

	Before rehabilitation program	During rehabilitation program	*P*
Number of participants	39	39	
**Caloric expenditure/week (kcal/week)**			
in all exercise-related activities	3,793.9 ± 2,751.0	3,876.7 ± 2,714.7	0.9
in at least moderate intensity exercise-related activities	2,521.4 ± 1,766.2	2,693.0 ± 2,137.6	0.9
in “fast walk for exercise”	158.7 ± 348.6	528.4 ± 553.2	<0.005
**Frequency/week**			
of all exercise-related activities	22.0 ± 13.2	23.9 ± 9.1	0.3
of at least moderate intensity exercise-related activities	8.8 ± 7.1	11.3 ± 5.8	<0.001
of walk for exercise	0.8 ± 1.6	4.6 ± 1.8	<0.0001
**Percent of participants**			
who exercises with moderate intensity	89.7	100	<0.05
who walk for exercise	28.2	100	<0.001

### Gait Parameters

Results on the 6-min walk tests taken pre- and post-intervention without auditory stimulation are reported in Table 1. An improvement was observed in distance, cadence, velocity, and stride length.

## Discussion

In this study, we demonstrated that our musical application (BeatWalk) for individualized gait rehabilitation in PD is a safe, user-friendly, and enjoyable solution displaying good observance. Practicing walking with it increases “walk fast” duration, confidence in gait, and quality of life. The application has been developed and tested first in a clinical setting, where it showed promising immediate effects on gait (Dotov et al., 2019). Using it at home in the context of a rehabilitation program was a new challenge, especially for patients with PD, who are mostly older adults and not always familiar with mobile technologies. Others have tested rhythmic cueing in rehabilitation in hospital (Dalla Bella et al., 2017) or at home but in the presence of a physiotherapist (Nieuwboer et al., 2007). Other studies were “mixed,” with a part of the treatment performed at the hospital and another part at home without a physical therapist (e.g., Murgia et al., 2018). Our program differs from these protocols, as patients used the BeatWalk application alone at home and most of them managed to use it successfully, thanks to the adapted interface (Garzo et al., 2018). More than 75% of the patients found the application easy to use and would recommend it to a friend.

The rehabilitation program using BeatWalk motivated patients to go outside, walk alone, and enjoy physical activity for almost eight and a half hours. Our patients’ caloric expenditure, already quite high at baseline (Stewart et al., 2001), did not significantly increase during physical activity but rather reorganized so as to privilege “walk fast for exercise.” This is probably more beneficial for the evolution of their disease.

The patients enjoyed performing physical activity with BeatWalk, a positive effect that was sustained for 20 sessions of gait rehabilitation. Combined with the personalized choice of the music genre, selecting music instead of a metronome for the stimulation might account for the patients’ enjoyment ratings. Listening to music is an enjoyable and rewarding activity (Sihvonen et al., 2017), an effect mediated by the dopaminergic system (Ferreri et al., 2019). Enjoyment is both a predictor and outcome of physical activity participation (Dacey et al., 2008). Expected enjoyment from physical activities can increase exercise intentions, adoption, and maintenance (Dunton and Vaughan, 2008).

We recently reported that impaired rhythmic skills are associated with a poor immediate response to rhythmic cues (Cochen De Cock et al., 2018) and to a less pronounced longer-term effect of cueing in a rehabilitation protocol (Dalla Bella et al., 2017). The music delivered by BeatWalk is modified in real-time so as to temporally adjust the musical beats to the patients’ steps. This interactive cueing was designed with the flexibility needed to induce synchronization in all patients, regardless of their rhythmic abilities. The spontaneous nature of entrainment implies that cognitive effort and deleterious effects of dual-tasking are minimized for patients. Interestingly, BeatWalk did not lead to a rise in the number of falls. The reduced double-tasking might also be part of this safety result. BeatWalk’s response to gait information is twofold: (1) tempo is tailored to patients’ cadence and (2) individual musical beats tend to slightly anticipate the footfalls. This subtle anticipation has a motivating effect and is part of how participants are induced to accelerate (Dotov et al., 2019).

BeatWalk delivers verbal feedback during the session. Participants also can follow their progress on the application screen. At the end of each session, the distance traveled and the mean speed of the latest and previous sessions were posted. This feedback has an important rewarding effect for patients and motivates physical activity (Ginis et al., 2016, 2017). Moreover, it could reduce fatigue, one of the main limiting effects of physical activity in patients with PD. Interestingly, in our study, the use of BeatWalk did not increase fatigue and patients completed almost all their sessions.

Fear of falling is common in PD (Lindholm et al., 2014). Self-efficacy, or the personal belief regarding the ability to perform a particular activity in a given situation, is an important factor of engagement in physical activity (Bandura, 1977). Fear of falling has already been identified as a barrier to physical exercise in PD (Ellis et al., 2013). Here, we demonstrated that patients who exhibited low observance to BeatWalk were those with increased fear of falling at baseline: fear of falling reduced the engagement in gait rehabilitation. Patients with a relatively high level of autonomy, probably felt more comfortable in using this tool. On the other hand, we also demonstrated that BeatWalk significantly reduced fear of falling and thus could facilitate exercise. Patients with increased fear of falling at baseline could benefit from gait rehabilitation with a physiotherapist prior to using BeatWalk. As such, the use of BeatWalk could initiate a virtuous circle for exercise and especially gait rehabilitation.

An increase in quality of life is another potential effect of the present intervention strategy. Physical activity is frequently associated with an improvement of quality of life in PD (Song et al., 2017), which has also been described in another gait rehabilitation program at home (Nieuwboer et al., 2007).

Another potential benefit of the present intervention strategy was not intended originally but became apparent with the recent sanitary crisis with COVID-19 pandemic infection. The requirements for confinement emphasized the need for auto-rehabilitation programs at home. Other intervention forms that may reduce impairments and improve quality of life are physiotherapy, occupational therapy, and speech therapy. Yet, these interventions require weekly sessions, impose stricter time and organizational constraints, and are not in compliance with current confinement restrictions, all of which represent a burden for the caregiver and the patients.

We observed non-controlled but encouraging results of efficacy on gait parameters in line with those observed in controlled studies by others (Nieuwboer et al., 2007; Dalla Bella et al., 2017) improving gait in the absence of stimulation.

This improvement can be the result of the increased physical activity associated with BeatWalk, supporting an improvement in muscular and cardiovascular capacities. It can also be the result of a restoration of the gait rhythmicity by the repeated use of music cueing, activating compensatory cerebello-thalamo-cortical loops, or increasing the activity of basal ganglia-thalamo-cortical circuits (Damm et al., 2019). Are these effects due to intrinsic motivational aspects of music, the rewarding effect of the interactive beat, or the constraints of a rehabilitation program, or a synergistic combination thereof? As encouraging as these results are, a large-sample randomized study currently in progress will compare walking in the absence of cueing (effect of physical activity), walking with music but no interactive cueing (motivating aspect of music), and walking with interactive music (reduced double-tasking and activation of the reward prediction mechanism).

## Conclusion

BeatWalk is a new wearable application increasing “walk for exercise” in a generally sedentary population. The application delivered music interactively synchronized to the patients’ gait and proved to be safe, well tolerated, easy to use, and enjoyable. BeatWalk appears as a very promising tool for implementing music technology solutions for health care in patients with movement disorders.

## Data Availability Statement

The raw data supporting the conclusions of this article will be made available by the authors, without undue reservation.

## Ethics Statement

The studies involving human participants were reviewed and approved by National Ethics Committee (CPP Sud Méditérannée III, Nîmes, France, ID-RCB: 2015-A00531-48). The patients/participants provided their written informed consent to participate in this study.

## Author Contributions

The study was conceived and planned by VC, DD, FG, MP, VD, ED, BB, and SB. VC, DD, PI, FG, CL, AGi, VD, and CG collected the data. VC, SL, and SB analyzed and interpreted the data, and were involved in the initial drafting of the manuscript. VC, DD, LD, FG, MP, CG, AGa, EH, ML, ED, RV, BB, and SB revised the paper. All authors contributed to the article and approved the submitted version.

### Conflict of Interest

The authors declare that the research was conducted in the absence of any commercial or financial relationships that could be construed as a potential conflict of interest.
